# Effects of attachment-based compassion therapy (ABCT) on brain-derived neurotrophic factor and low-grade inflammation among fibromyalgia patients: A randomized controlled trial

**DOI:** 10.1038/s41598-019-52260-z

**Published:** 2019-10-30

**Authors:** Jesus Montero-Marin, Laura Andrés-Rodríguez, Mattie Tops, Juan V. Luciano, Mayte Navarro-Gil, Albert Feliu-Soler, Yolanda López-del-Hoyo, Javier Garcia-Campayo

**Affiliations:** 1 Primary Care Prevention and Health Promotion Research Network (redIAPP), Madrid, Spain; 2 Institut de Recerca Sant Joan de Déu, Esplugues de Llobregat, Spain; 3 Teaching, Research & Innovation Unit, Parc Sanitari Sant Joan de Déu, St. Boi de Llobregat, Spain; 4 Unitat de Psicologia Bàsica, Facutat de Psicologia, Universitat Autònoma de Barcelona, Barcelona, Spain; 5 Department of Clinical, Neuro & Developmental Psychology, Vrije Universiteit, Amsterdam, Netherlands; 6 Department of Psychology and Sociology, University of Zaragoza, Zaragoza, Spain; 7 Instituto de Investigación Sanitaria de Aragón (IISA), Hospital Universitario Miguel Servet, Zaragoza, Spain

**Keywords:** Psychology, Biomarkers

## Abstract

Fibromyalgia (FM) is a disabling syndrome characterized by chronic pain associated with fatigue. Its pathogenesis is unknown, but alterations in central sensitization, involving an imbalance of brain-derived neurotrophic factor (BDNF) and inflammatory biomarkers, appear to be implicated. The aim of this study was to evaluate the impact of attachment-based compassion therapy (ABCT) on levels of BDNF, the inflammatory markers TNF-α, IL-6, IL-10, and the C-reactive protein (CRP), analysing whether biomarkers play a mediating/moderating role in improvements in FM functional status. Thirty-four female patients with FM participated in a RCT and were assigned to ABCT or relaxation therapy. Blood extractions were conducted at baseline and post-intervention, with self-report assessments of functional status (FIQ) at baseline, post-intervention and 3-month follow-up. A pro-inflammatory composite was obtained by summing up IL-6, TNF-α and CRP normalized values. Non-parametric tests, analysis of variance and regression models were used to evaluate treatment and mediation/moderation. Compared to relaxation therapy, ABCT showed significant improvements in FIQ and decreases in BDNF, CRP, and pro-inflammatory composite. Changes in BDNF had a mediating role in FIQ. ABCT seems to reduce BDNF and appears to have anti-inflammatory effects in FM patients. Reductions in BDNF could be a mechanism of FM functional status improvement.

**Clinical Trial Registration:** http://ClinicalTrials.gov, identifier NCT02454244. Date: May 27th, 2015.

## Introduction

Fibromyalgia (FM) is a debilitating rheumatic chronic pain syndrome of unknown and complex aetiology, which includes symptoms such as widespread pain, fatigue, disturbed sleep, cognitive difficulties, psychological distress and associated affective disorders^1–3^. Its pathogenesis is not clearly understood, and, up to now, no curative treatment for this condition has been found^4^. The central nervous system (CNS) might play a leading role^5^, involving possible altered pain pathways in the form of central sensitizations and abnormal amplifications of pain^4,6^. It has been said that ongoing peripheral nociceptive input seems to be important to maintain central sensitization^7^. In this sense, FM has been related to a variety of physical and/or psychological stressors, which may contribute to a chronic pro-inflammatory state (both in the CNS and in peripheral tissues), which in turn could also exert effects in central processing of pain^8^. These possible altered pain pathways might involve low-grade baseline chronic neuro-inflammation processes, with stress peptides triggering the release of neurosensitizing mediators^9^.

Several biological mechanisms have been proposed in the development of the altered state in FM. Among others, higher levels of pro-inflammatory cytokines (e.g. interleukin IL-6 and Tumour Necrosis Factor or TNF-α) as well as lower levels of anti-inflammatory cytokines (e.g. interleukin IL-10) have been consistently observed in the central sensitized state of FM^8,10–12^. In general, it has been argued that an imbalance of pro- and anti-inflammatory cytokines could lead to a chronic inflammatory status in the CNS and the peripheral nervous system ‒ i.e. neuro-inflammation ‒ thus facilitating the sensitization of peripheral nerves to nociceptive stimuli^8,11^. Moreover, cytokines also affect neurotransmitters in the CNS involved in the experience of pain^13^ as well as the prolonging of other FM-related symptoms, such as fatigue, sleep problems, cognitive disturbances, and affective disorders^8,13^. Levels of C-reactive protein (CRP) have also been found to be higher in FM patients^14^. CRP is an acute phase protein elevated in inflammatory contexts stimulated by cytokines^15^, and it has been significantly correlated with IL-6 levels, reinforcing the idea that inflammation may contribute to FM symptoms^14^. In addition, positive correlations between increased brain-derived neurotrophic factor (BDNF) and S100B protein levels ‒ which are associated with the coexistence of central sensitivity and lower pressure pain thresholds in FM ‒ have recently been identified^16^. BDNF is known to play a key role in a variety of neuroplasticity processes, including pain modulation, pain transduction, nociception, and hyperalgesia^17^, all of which are altered in FM. Plasma levels of BDNF have also been found to be increased in patients with fibromyalgia^18^. Thus, BDNF provides a possible promising first step towards the development of new diagnostics of FM based on biomarkers^19^. In summary, an imbalance of pro- and anti- inflammatory cytokines as well as higher levels of CRP and BDNF have been observed in the plasma and/or serum of FM patients^4,8,12^. It has been proposed that the baseline pro-inflammatory status of chronic pain patients might have a moderating role on treatment improvements. Specifically, higher pre-treatment levels of IL-6 and TNF- α have been found to be associated with reduced improvement in pain intensity and other psychological health-related outcomes, providing a possible explanation of the heterogeneity in treatment response^20^.

The absence of consensus for curative treatments of FM^21^ and its large presence in the general population ‒ with global prevalence values of approximately 2.7%^4^ ‒ together with resulting unemployment, number of days of absenteeism from work and incapacity benefits claimed^22^ has motivated a large amount of research in this area. Few pharmacological treatments have demonstrated a significant impact on multiple FM symptom domains, but non-pharmacologic interventions ‒ e.g. psychological cognitive behavioural therapy ‒ have demonstrated multidimensional effects^23^. That is why, in the last few years, so-called “third-wave psychological interventions”, which seek to activate values and foster interpersonal virtues such as compassion, forgiveness and gratitude^24^, have been an object of special focus and attention^25^. For instance, Montero-Marin *et al*.^26^ recently found that a new third-wave psychological treatment, attachment-based compassion therapy (ABCT) ‒ which is composed of a regimen of 8 sessions of 2 hours of mindfulness and compassion practices ‒ administered as coadjutant treatment along with standard of care, improved in a significant and clinically relevant way the general health status of patients with FM compared to a suitable active control group of relaxation therapy. So far, there is some evidence of the regulation of and changes in the levels of inflammatory cytokines and in the expression of the corresponding genes as a result of mindfulness practices in distinct populations, including experienced meditators^27–30^, suggesting that the regulation of inflammatory pathways may configure some of the mechanisms underlying the therapeutic potential of this type of interventions. However, no studies have investigated the influence of the specific ABCT programme ‒ which also adds compassion practices ‒ on BDNF and low-grade inflammation level of FM patients.

With this background, the main aim of the present exploratory study was to extend previous clinical findings reported by Montero-Marin *et al*.^26^ by analysing the possible influence of ABCT (vs relaxation therapy) on the levels of BDNF and CRP and on the levels of IL-6, TNF-α and IL-10 cytokines in patients suffering from FM. In addition, we also aimed to analyse whether the referenced biomarkers play a mediating and/or a moderating role on clinical improvements in the FM functional status. Our general exploratory hypotheses where that: a) ABCT would result in significantly greater decreases in the BDNF and pro-inflammatory markers as well as significantly greater increases in the anti-inflammatory markers compared with the relaxation condition; and b) BDNF and (pro- and anti-) inflammatory biomarkers would play a significant mediating and moderating role on clinical improvements in the FM functional status.

## Method

A parallel randomized controlled clinical trial (RCT) design was used. Through two arms (ABCT vs relaxation therapy) and pre-treatment, post-treatment, and 3-month follow up assessments, the present work reports secondary data that form part of a larger study^26^ examining the efficacy of ABCT in the treatment of FM patients (TrialTrials.gov Registration: NCT02454244). Findings from a third treatment arm outlined in the protocol that used mindfulness plus amygdala retraining will be reported elsewhere.

### Procedure

Study participants were recruited from eight primary health-care centres in the city of Zaragoza, Spain. The present study inclusion criteria were: (1) male or female aged between 18 and 65 years, (2) able to read and understand Spanish, and (3) diagnosed with FM criteria (ACR 1990) by a rheumatologist working for the Spanish National Health Service (SNHS). The exclusion criteria were: (1) aged <18 or>65 years, (2) presence of a severe Axis I psychiatric/somatic disorder, autoimmune disease, or use of corticosteroid medication, and (3) current participation in another clinical trial. Other medication use was permitted as part of standard care as long as the participant agreed not to change the dosage during the study period.

General practitioners (GPs) identified potential participants who were then interviewed at the same clinic by an independent researcher until the required sample size was achieved. Those patients who met the inclusion/exclusion criteria and gave their written informed consent before the baseline assessment were included in the study and randomized. A researcher who had no involvement in the study generated a simple random allocation sequence by using computer software to determine group assignment. Randomization was implemented via telephone, and the allocation details were concealed from the other researchers involved until all participants had been assigned. Participants were not informed of group allocation until after completion of baseline assessments, and they were not informed which allocation condition was the target intervention. The outcome assessor remained blind as to participant allocation.

Informed consent was obtained from February to April 2015, randomization occurred in April 2015, interventions were delivered from May to October 2015, and follow-up measurements were collected from October 2015 to March 2016. The RCT was conducted according to the “Initiative on Methods, Measurement and Pain Assessment in Clinical Trials” (IMMPACT) recommendations and the “Consolidated Standards of Reporting Trials” (CONSORT) guidelines. The study protocol was approved by the ethical review board of the regional health authority of Aragon (CEICA), Spain (PI15/0049; 01/04/2015). A more detailed explanation of the procedure is published elsewhere^26^.

### Participants

Sample size estimation for the RCT primary outcome of FM functional status (Fibromyalgia Impact Questionnaire, ‒ FIQ) was based on a clinically relevant expected difference of at least 20%. A previous study with similar features found a FIQ mean and SD of 70.8 and 15.2, respectively^31^. Therefore, a difference of 14.6 points between groups was our target (which is equivalent to 0.95 SDs). To detect this difference with a 5% significance level and 80% statistical power in a two-tailed test, we needed 18 participants per group. We expected a dropout rate of approximately 20%, so we increased the numbers to reach an initial total sample size of 42 subjects.

Finally, a total of 34 FM patients (ABCT: *n* = 19; relaxation: *n* = 15) who completed all of the biomarker measurement tests were included in the present study. The distributions of socio-demographic and clinical characteristics of participants at baseline for each group are shown in Table 1. Participants were all female, in their early fifties, mostly with partner, and dwelling in their own home in an urban residence. They were roughly equally distributed among primary, secondary and university education levels, and the majority were in sick-leave/disability, with a median income level of 1–2 minimum inter-professional salaries ‒ MIS − (*Q*_1_ = <MIS − *Q*_3_ = 2–4 MIS). In clinical terms, participants presented a mean of FM symptoms above the average of FM patients [FIQ: *Mn* = 63.91 (*SD* = 18.09)]. They had been suffering from FM for a mean of 40 months (SD =30.64), they visited mental health services a median of 2 times during the previous year (*Q*_1_ = 0 − *Q*_3_ = 8), and 30 of them were taking FM-related medication (88.2%). There were no significant differences between groups in any of the referenced variables (Table 1).

**Table 1:** Characteristics of participants at baseline.

Variables	ABCT (*n* = 19)	Relaxation (*n* = 15)	*p*
**Socio-demographic**			
Age, *mean* (*SD*)	52.63 (7.76)	53.60 (5.08)	0.679
Sex female, *n* (%)	19 (100)	15 (100)	1.00
Stable relationship, *n* (%)	16 (84.2)	11 (73.3)	0.672
Homeowner, *n* (%)	18 (94.7)	14 (93.3)	0.999
Urban residence, *n* (%)	15 (84.2)	15 (100)	0.113
Education level, *n* (%)			0.123
Primary school	9 (47.3)	2 (13.4)	
High school	6 (31.6)	8 (53.3)	
University	4 (21.1)	5 (33.3)	
Employment status, *n* (%)			0.349
Looking after the family/home	7 (36.8)	3 (20.0)	
Employed	1 (5.3)	4 (26.6)	
Sick leave/disabled	9 (47.4)	7 (46.7)	
Unemployed	2 (10.5)	1 (6.7)	
Income level, median (*Q*_1_*‒Q*_3_)	1-2 MIS	1-2 MIS	0.851
	(<MIS ‒ 2-4 MIS)	(1-2 MIS ‒ 1-2 MIS)	
**Clinical variables**			
Fibromyalgia (FIQ), *mean* (*SD*)	67.31(17.17)	61.12 (20.21)	0.341
Range (0-100)			
Months with FM symptoms, *mean* (*SD*)	40.40 (29.00)	43.13 (33.20)	0.800
Visits to MHS last year, *median* (*Q*_1_*‒Q*_3_)	2 (0–11)	1 (0–4)	0.560
On medication last year, *n* (%)	18 (94.7)	12 (80.0)	0.299
Type of medication last year, *n* (%)			
Analgesics*	6 (31.6)	4 (26.7)	0.999
NSAIDs	7 (36.8)	3 (20.0)	0.451
Anxiolytics	5 (26.3)	7 (46.7)	0.288
Opiates	8 (42.1)	3 (20.5)	0.271
Antidepressants	10 (52.6)	8 (53.3)	0.999
Hypnotics	4 (21.1)	0 (0.0)	0.113
Anticonvulsants	6 (31.6)	3 (20.0)	0.697

*Note*: *Means* with *standard deviations* (*SD*), *medians* with *interquartile range*, or *number of cases* with *percentages* (%) for each group are presented where appropriate. MIS: minimum inter-professional salary. MHS: mental health services. *Painkillers in a broad sense, including triptans or anti-migraine drugs (e.g. Paracetamol, Metamizole, Sumatriptan, Rizatriptan).

### Treatments

Both ABCT and relaxation were presented as suitable active interventions and included 8 weekly 2-hour sessions and 3 monthly booster sessions (2 hours each) that were combined with treatment as usual (TAU) provided by the SNHS for FM patients. TAU was offered by the corresponding general practitioner and consisted of administering drugs for pain as well as antidepressants ‒ it could also include pharmacological treatments for insomnia and fatigue. Daily homework assignments with specific exercises (i.e. meditation or relaxation) were recommended.

#### Attachment-based compassion therapy (ABCT)

The ABCT training program^32^ was especially adapted for FM patients^26^. In general, this intervention involved exercises of mindfulness and visualizations focused on augmenting the patients’ ability to be considerate and kind towards themselves as well as others’ experiences when facing suffering. Specifically, ABCT includes exercises of mindfulness breathing, compassionate body scan, connecting with affection and compassion with others, replacing the inner critical voice with a more tolerant one, awareness of the ability to receive affection and of one’s own capacity to give affection and forgiveness and to manage envy and others’ suffering in difficult relationships, equanimity and gratitude.

#### Relaxation therapy

The relaxation arm constituted a low-intensity and non-specific but suitable active control condition that included different relaxation techniques^26^, such as guided relaxation through imagery, relaxation through landscape visualizations, working with emotions through imagination, autogenic training by imagining a ball of light with sensations of heat and body sweeping heaviness to facilitate relaxation, progressive muscle relaxation by tensing and relaxing the muscles to become aware of the different sensations and to reach relaxation, and different breathing exercises, such as slow and deep inspiration and exhalation to calm anxiety^26,33^.

### Study measures

Participants completed a socio-demographic paper-and-pencil survey at baseline that included: age, sex, marital status (in a stable relationship vs not in a stable relationship), dwelling (homeowner vs renter), place of residence (urban vs non-urban), education (Primary, High school, University), employment status (looking after the family/home, employed, sick leave/disabled, unemployed), and income level (less than minimum inter-professional salary or <MIS, 1‒2 MIS, 2‒4 MIS, >4 MIS). Information was collected on the number of months participants had suffered from FM, the number of visits to mental health services during the last year, whether they were taking FM-related medication during the last year and which ones (analgesics, NSAIDs, anxiolytics, opiates, antidepressants, hypnotics, and anticonvulsants), the credibility of their intervention (on a scale from 0 to 10), and their treatment preference (ABCT, relaxation, other, indifferent).

The *Fibromyalgia Impact Questionnaire* (FIQ)^34^, which is the gold-standard measure of functional status in FM patients, was carried out at baseline, post-treatment and 3-month follow up as the primary outcome measure of the trial. It is a self-report measure based on 10 items that were developed to capture a broad spectrum of problems and difficulties related to FM (e.g. ability to perform physical activities, subjective feelings, work ability, pain, fatigue, morning tiredness, stiffness, anxiety, and depression). It is considered to be a primary efficacy end-point measure of response to therapy, and it is scaled from 0 to 100, with higher scores indicating greater functional impairment. The FIQ has demonstrated good psychometric properties in its Spanish validation^35^.

Participants were scheduled for blood extraction prior to treatment (forming part of the baseline evaluation) and some days after treatment (post-treatment evaluation, which was within 5 days following treatment in all of cases). To minimize circadian variability in immunological markers, all blood samples were collected between 8:00–8:30 AM after night fasting. After extraction, blood was centrifuged and serum was frozen to −80 °C until the analyses at LABCO laboratories, where BDNF was analysed with ELISA (R&D systems^©^), IL-6, TNF-α, and IL-10 with Immulite^©^ 1000 (Siemens), and CRP with immunoturbidimetry (CRP Beckman Coulter^©^). The kits were used, and the levels of biomarkers calculated according to the manufacturer instructions. The parallel design of the study ensured that the groups would display equivalent seasonal variability.

### Statistical analyses

We used means (*SD*), medians (interquartile range), and frequencies (percentages) to describe the distribution of baseline characteristics across arms according to the nature of each variable ‒ the corresponding *t*-test, Mann-Whitney *U*, and Fisher exact probability test were used for comparisons between the ABCT and relaxation groups.

All outcome measures were evaluated for normality with the Kolmogorov-Smirnov test. Concentrations of cytokines and CRP measures were subjected to a natural logarithmic transformation to normalize the significantly skewed data distributions (FIQ and BDNF showed a normal distribution, so they were always analysed without transformation). In addition, to obtain a general view of the inflammatory status, a composite index score (General Inflammation ‒ GI composite) integrating those biomarkers that are theoretically of pro-inflammatory type^8,15^ was calculated by adding the corresponding *Z* scores for the normalized IL-6, TNF-α and CRP values. This inflammation score allows for the calculation of an aggregate index of all pro-inflammatory biomarkers by converting them to a comparable score and then totalling the values for each individual^36^. This procedure was contrasted by a principal-component analysis of the referred established measures of pro-inflammatory activity^8,15^, identifying one single factor at pre-test (accounting for 44% of the variance, with the factor loadings of IL-6 = 0.70, TNF-α = 0.62, and CRP = 0.68) and at post-test (43% of the variance, with the loadings of IL-6 = 0.76, TNF-α = 0.51, and CRP = 0.69). In this context, a composite index takes advantage of the predictive value of the three pro-inflammatory measures considered while minimizing measurement errors of the single components^37^, so the use of that index was chosen. Previous studies have designed and used similar comprehensive indices of inflammation that combine some interleukins and/or other biomarkers of inflammation plus CRP^36–38^.

First, we examined the effect of ABCT compared with the relaxation condition on FM functional status (FIQ) and all of the biomarker variables by an intention-to-treat basis and using the non-parametric Mann-Whitney U test to compare the two groups on the raw (not log-transformed) delta-values (change scores). Violin plots representing both information pertaining to the summary statistics of the data set as well as the density of data points throughout the range of the raw data set were used to represent treatment effects. After carrying out log transformations (where necessary), we also conducted repeated measures (RM) ANOVAs to introduce the possibility of calculating partial eta squared effect sizes measures (*ƞ*^2^) and ANCOVAs at post-treatment (and at follow up on FIQ), with the baseline as a covariate, to gain statistical power and increase confidence in our conclusions^39,40^. Partial eta squared is interpreted as follows: *ƞ*^2^ ≤ 0.01 are small, *ƞ*^2^ = 0.06 are intermediate, and *ƞ*^2^ ≥ 0.14 are large^41^. Descriptive statistics (*Means* and *SD*s) and the percentages of pre-post increment (*Δ%*) of biomarkers were also calculated.

Following Judd, Kenny & McClelland^42^, the potential mediating and moderating effects of biomarkers on the FM functional status were evaluated using the total group of participants as a within-subjects design to achieve a more powerful analysis by regressing the FM functional status pre‒follow up difference on the biomarker pre‒post difference and sum using ordinary least squares (OLS) regression models. This approach proposes a test of mediation and moderation for designs where the same individuals are measured on distinct time points, including assessments of the mediator/moderator and the dependent variable. It is applicable whenever observations are non-independent of each other because they come from the same participant, and it includes designs in which the factor that causes the dependence is crossed with the independent variable, which is the repeated-measures factor. In these conditions, it is proposed that mediation is indicated when the difference in the dependent variable depends on the difference in the mediator/moderator, whereas moderation is indicated if the difference in the dependent variable depends on the sum of the mediator/moderator variable^42^. Other works have explored the analysis of mediation/moderation in within-subjects designs using change scores^43,44^, but the Judd *et al*. approach^42^ has been widely used and applied in previous research^45–54^ because it is the dominant method for assessing mediation/moderation in within-subjects designs^55^, and it has even been referred to as an important and valuable contribution by authors from other different analytical perspectives^56^.

**Figure 1:**
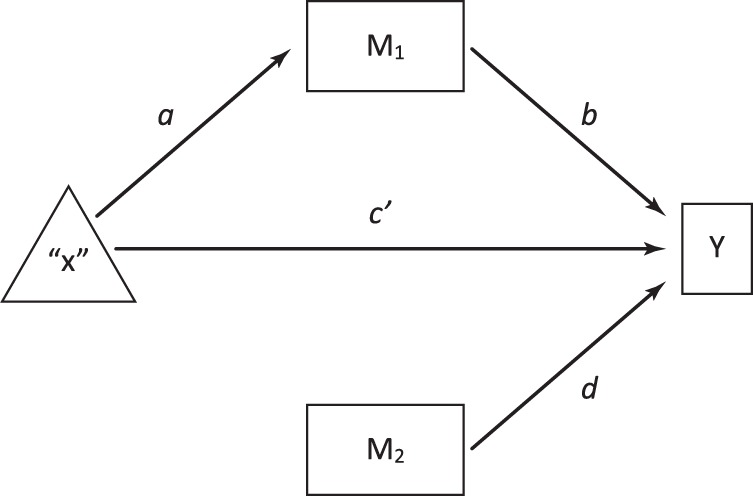
Generic path diagram of a mediation/moderation within-subjects model*. *From the proposal of Judd, Kenny and McClelland (2001) and the development of Montoya and Hayes (2017). The independent variable is the repeated-measures factor (“X”). M_1_ is the pre-post difference (mediating effect). M_2_ is the pre-post sum (moderating effect). The FM functional status pre-follow up difference is the dependent variable (Y). “*a x b”* = indirect effect. *c*′ = direct effect after adjusting for the mediating effect*. d* = moderating effect.

Nevertheless, to facilitate the understanding of readers and to produce a more powerful analysis based on an indirect effect test using a bootstrapped confidence interval, we used the path analysis framework that was recently developed for within-subjects designs by Montoya & Hayes^55^ from the Judd *et al*. approach^42^. For that, we explored the indirect relationships among the repeated-measures factor, the biomarker pre‒post difference and sum and the FM functional status pre‒follow up difference, using OLS analysis with unstandardized path estimates from regression coefficients, where: (i) the repeated-measures factor was the independent variable (“X”), (ii) the biomarker pre‒post difference (M_1_) and sum (M_2_) were the mediating and moderating factors respectively, and (iii) the FM functional status pre‒follow up difference was the dependent variable (Y). We introduced the centred biomarker sum to assess the residual FM functional status over and above the mediating effect by means of the intercept^42,55^ as follows: *Y* = *c*′ + *b (M*_1_*)* + *d [(M*_2_*)* − *(mean M*_2_*)]* + *e*; where *c*′, *b* and *d* are the regression intercept and slopes respectively, and *e* denotes the error in estimation. A generic path diagram of a mediation/moderation within-subjects design from the proposal of Judd *et al*.^42^ and the development of Montoya & Hayes^55^ can be observed in Fig. 1. Beta coefficients (*β*) were used to assess the individual contribution of the biomarker pre‒post difference and sum as predictors to explain the FM functional status pre‒follow up difference, and the Wald test was used to evaluate their statistical significance. Additionally, the FM functional status pre‒follow up difference was regressed using the stepwise method on the pre‒post difference scores of all biomarkers that showed possible mediating effects in the Judd *et al*. formulation^42^ (*b* significant in Fig. 1). The regression coefficient for bootstrapped indirect effects was calculated, as was its 95% confidence interval (95% CI). This test can be applied to small samples to overcome possible problems of asymmetry in the distribution of the indirect effects^57^, which are statistically significant when the 95% CI of the corresponding “*a x b”* parameter does not include zero. Multiple determination coefficients (*R*^2^) were calculated to observe the explanatory power of the regression models. Finally, tolerance (*T*) and variance inflation factor (*VIF*) were calculated to evaluate the degree of collinearity among possible mediators. Values of *T* < 0.10 and *VIF* > 10 represent unacceptable levels of *T* and *VIF* ^58^.

The overall *α* level for the primary outcome analyses of FM functional status (FIQ) ‒ which presented three possible comparisons, as was registered in ClinicalTrials.gov (NCT02454244) and published in a previous paper^26^ ‒ was set at0.05 using two-tailed tests and Bonferroni’s multiple comparisons criterion. However, in the present secondary analysis of biomarkers, we did not make an adjustment for multiple tests by means of Bonferroni correction. This adjustment is usually used assuming that all tests are independent of each other. However, in some practical applications, such as in this case, that is often not maintainable. In addition, it is well-known that Bonferroni correction can be too conservative, causing a high rate of false negatives, which in turn contributes to publication bias^59^. Thus, considering all of the above and keeping in mind that the present work is a secondary analysis of a very exploratory nature^60^, no corrections for multiple measurements were used for the biomarker outcomes.

Statistical analyses were carried out using the IBM SPSS Statistics for Windows, Version 19.0. Armonk, NY: IBM Corp.

### Ethical approval

The study was approved by the ethical review board of the regional health authority, Aragon Ethical Committee (CEICA), Spain (PI15/0049; 01/04/2015). All procedures performed in this study involving human participants were in accordance with the ethical standards of the institutional research committee (Aragon Ethical Committee, CEICA), the 1964 Helsinki Declaration and its later amendments or comparable ethical standards, and the Declaration of Madrid of the World Psychiatric Association and Uniform Requirements for Manuscripts Submitted to Bio-Medical Journals. Informed consent was obtained from all individual participants included in the study.

## Results

**Figure 2:**
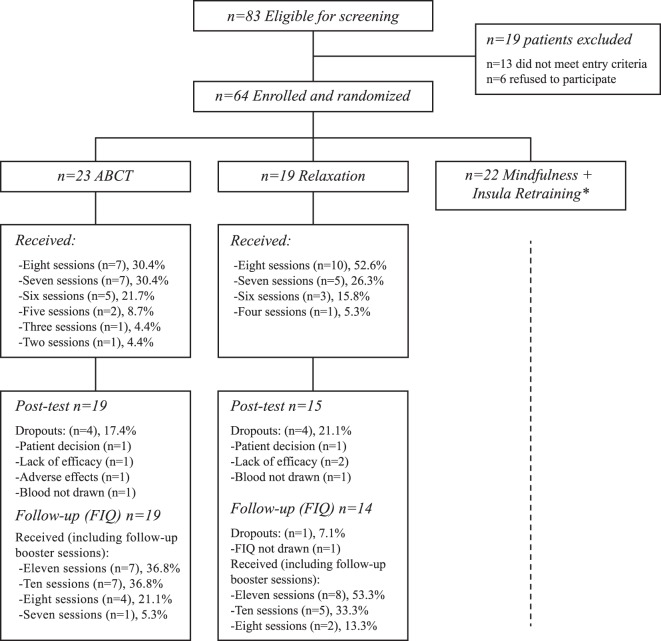
Flowchart of the study. *For space reasons, the results of the “Mindfulness + Insula Retraining” arm will be detailed elsewhere.

The study flowchart is provided in Fig. 2. Of the 83 patients who were eligible for screening, 19 were excluded. The reasons for exclusion were (a) not diagnosed by a rheumatologist (*n* = 8), (b) older than 65 years (*n* = 2), and (c) suffering from a psychiatric disorder (schizophrenia; *n* = 1) or severe medical disorder (lupus; *n* = 2). Six patients refused to participate. Of the 64 participants who were eligible and randomly allocated to a treatment group, 23 were assigned to ABCT and 19 to relaxation (22 participants were assigned to the ‘mindfulness + amygdala retraining’ group, the results of which will be reported elsewhere). The measurement completion rate was high, with 34 (81.0%) participants completing all of the biomarker outcomes. Eight participants dropped out of the study (4 in the ABCT group: 1 patient decision, 1 lack of efficacy, 1 low-grade adverse effect, 1 blood not drawn; and 4 in the relaxation group: 1 patient decision, 2 lack of efficacy, 1 blood not drawn). Therefore, a total of 19 (82.6%) participants in the ABCT, and 15 (78.9%) in the relaxation group completed the study (Fisher’s test, *p* = 0.999) and were included and analysed as they were originally assigned. The number of sessions attended, including the booster reminder assistances, was similar between groups (ABCT: *Median* = 10 sessions; *Q*_1_–*Q*_3_ = 8–11; relaxation: *Median* = 11 sessions; *Q*_1_–*Q*_3_ = 10–11; *Z* = 1.12; *p* = 0.302). All participants who were included in the analyses completed >50% of sessions, and 33 participants (97.1%) completed ≥75% of sessions. There were no differences between groups in terms of either credibility (ABCT: *Median* = 8; *Q*_1_–*Q*_3_ = 8 ‒ 8; relaxation: *Median* = 8; *Q*_1_–*Q*_3_ = 7‒8; *Z* = 0.88; *p* = 0.515), or treatment preference [ABCT group: ABCT = 4 (21.1%), relaxation = 2 (10.5%), other = 3 (15.8%), indifferent = 10 (52.6%); relaxation group: ABCT = 4 (26.7%), relaxation = 1 (6.7%), other = 3 (20.0%), indifferent = 7 (46.7%); Fisher test *p* = 0.956].

Although participants agreed not to change the dosage of any prescribed medication during the study period, we detected a pre-post variation in the frequency of opioid consumption: pre-ABCT = 8 (42.1%), post-ABCT = 6 (31.6%); pre-relaxation = 3 (20.5%), post-relaxation = 5 (33.3%). We applied Fisher’s exact test at pre- and post-test, with a result of p = 0.271 and p = 0.999 respectively, and thus the two groups were rather similar with regard to the number of opiate users. All but one opioid user (who used Fentanyl) were users of Tramadol. Opioid consumption (in mg) remained very similar in each group: a) the Mann-Whitney U test showed no significant differences in opioid consumption between groups either at pre-test (ABCT vs relaxation, Z = −1.23, p = 0.319) or at post-test (ABCT vs relaxation, Z = −0.15, p = 0.918), and the Wilcoxon test showed no significant differences between the pre-post ABCT group consumption (Z = −1.61; p = 0.110) and in the pre-post relaxation group consumption (Z =<0.01; p = 0.999). The Mann-Whitney U test for comparing the two groups on the delta-values (post‒pre) for opioid consumption showed non-statistically significant values (Z = −1.51, p = 0.319). Thus, the two groups were rather similar with regard to the number of opiate users and the amount of opiate consumption in mg at each time point.

### Effect of ABCT vs relaxation on FM functional status

The non-parametric Mann-Whitney U test on the FIQ delta-values showed significant differences between-groups at post-test (*Z* = −3.28; *p* = 0.001) and follow-up (*Z* = −2.48; *p* = 0.012). There was a significant ‘group x time’ interaction (RM ANOVA: *F*_(2,30)_ = 9.12; *p* = 0.001; *ƞ*^2^ = 0.38), with decreases in the FIQ favouring the ABCT condition at post-test (ANCOVA: *F*_(1, 31)_ = 14.51; *p* = 0.001; *ƞ*^2^ = 0.32) and follow up (ANCOVA: *F*_(1, 30)_ = 8.41; *p* = 0.007; *ƞ*^2^ = 0.22) [ABCT: pre-test *Mn* = 67.31 (*SD* = 17.17), post-test *Mn* = 43.11 (*SD* = 10.43), follow up *Mn* = 49.38 (*SD* = 15.44); relaxation: pre-test *Mn* = 59.60 (*SD* = 18.89), post-test *Mn* = 61.00 (*SD* = 25.78); follow-up *Mn* = 66.72 (*SD* = 17.90)].

### Effect of ABCT vs relaxation on BDNF and inflammatory biomarkers

**Figure 3:**
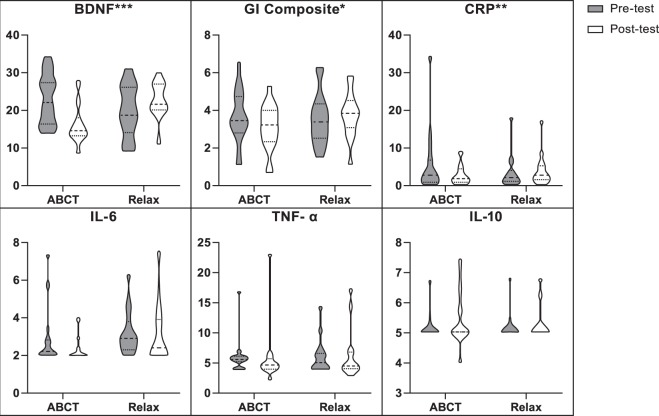
Effects of ABCT vs. Relaxation on BDNF and inflammatory biomarkers. Note: Graphics are violin plots. The scales are different in different sections to make them legible (ng/ml in BDNF; pg/ml in CRP, IL-6, TNF-α and IL-10; and a natural logarithm normalization sum in the GI composite). Raw data was used in all measures (except for the GI Composite, which was calculated by adding the Z scores for the normalized log transformed IL-6, TNF-α and CRP values) to provide a more informed visualization of the treatment effects. *p < 0.05. **p < 0.01. ***p < 0.001.

As seen in Table 2, the non-parametric Mann-Whitney U test on the raw (non-transformed) delta-values showed significant differences between-groups at post-test in BDNF (*Z* = −3.94; *p* < 0.001), GI composite (*Z* = −2.34; *p* = 0.019), and CRP (*Z* = −2.69; *p* = 0.006). In addition, significant ‘group x time’ interactions (RM ANOVA) were observed for BDNF (*F*_(1, 32)_ = 24.67; *p* < 0.001) with large effects (*ƞ*^2^ = 0.44); GI composite (*F*_(1, 32)_ = 5.29; *p* = 0.028) with large effects (*ƞ*^2^ = 0.14); and CRP (*F*_(1, 32)_ = 4.67; *p* = 0.038) with moderately large effects (*ƞ*^2^ = 0.13), all of them showing decreases in the ABCT group. However, no significant ‘group x time’ interactions for IL-6 (*F*_(1, 32)_ = 0.18; *p* = 0.675; *ƞ*^2^ = 0.01), TNF-α (*F*_(1, 32)_ = 1.26; *p* = 0.269; *ƞ*^2^ = 0.04) and IL-10 (*F*_(1, 32)_ = 0.03; *p* = 0.875; *ƞ*^2^  = < 0.01) were found. These results were also replicated using ANCOVAs at post-test after controlling for baseline scores (Table 2). Figure 3 is a graphic representation of the effects of ABCT vs relaxation on BDNF and low-grade inflammatory biomarkers.

**Table 2:** Between-group analyses on levels of biomarkers.

Outcomes	ABCT (*n* = 19)		Relaxation (*n* = 15)		Mann-Whitney U test		RM ANOVA			ANCOVA		
	*Mn* (*SD*)	*Δ%*	*Mn* (*SD*)	*Δ%*	*Z*	*p*	*F*_*(df)*_	*p*	*ƞ* ^2^	*F*_*(df)*_	*p*	*ƞ* ^2^
BDNF (ng/ml)												
*Pre*	23.03 (6.65)		19.78 (6.89)									
*Post*	16.34 (5.02)	−29.1	22.80 (4.69)	15.2	−3.94	<0001	24.68_(1,32)_	<0001	0.44	28.81_(1,31)_	<0001	0.48
GI composite*												
*Pre*	3.70 (1.40)		3.56 (1.39)									
*Post*	3.12 (1.18)	−15.7	3.75 (1.20)	5.3	−2.34	0.019	5.29_(1,32)_	0.028	0.14	6.28_(1,31)_	0.018	0.17
CRP (pg/ml)												
*Pre*	5.60 (7.95)		3.46 (4.42)									
*Post*	2.89 (2.56)	−48.4	4.07 (4.19)	17.6	−2.69	0.006	4.67_(1,32)_	0.038	0.13	4.63_(1,31)_	.039	0.13
IL-6 (pg/ml)												
*Pre*	2.90 (1.56)		3.18 (1.19)									
*Post*	2.39 (0.70)	−17.6	3.13 (1.60)	−1.6	−0.47	0.656	0.18_(1,32)_	0.675	0.01	2.90_(1,31)_	0.098	0.09
TNF-α (pg/ml)												
*Pre*	6.05 (2.75)		6.10 (2.87)									
*Post*	5.61 (4.33)	−7.3	6.23 (4.09)	2.1	−1.03	0.319	1.26_(1,32)_	0.269	0.04	1.26_(1,31)_	0.271	0.04
IL-10 (pg/ml)												
*Pre*	5.15 (0.41)		5.14 (0.46)									
*Post*	5.41 (0.86)	5.1	5.34 (0.59)	3.9	−0.37	0.758	0.03_(1,32)_	0.875	<001	0.02_(1,31)_	0.881	<0.01

*Mn*: mean. *SD*: standard deviation. *Δ%*: percentage increment. *Z*: Z-value. *p*: *p*-value. *F*: Snedecor’s *F. ƞ*^2^: partial eta squared as an effect size measure. Mann-Whitney U test: Non-parametric Mann-Whitney U test on the raw (non-normalized) delta-values for the biomarkers (post-pre values), except for the GI composite, which was calculated by adding the *Z* scores for the normalized IL-6, TNF-α and CRP values. RM ANOVA: Repeated measures analysis of variance with pre- and post-treatment measures. ANCOVA: analysis of covariance at post-treatment with the baseline measure as a covariate. ANOVA and ANCOVA analyses were performed on *log* values (except for the BDNF). *Descriptive values are presented as raw scores except for the GI composite, which is a natural logarithm normalization sum.

### Mediating and moderating effects of biomarkers on FM functional status

When the FM functional status pre‒follow up difference was regressed on the pre‒post biomarker difference (i.e. mediating effect) and sum (i.e. moderating effect), we observed that the BDNF difference was a significant predictor of the FM functional status (*β* = 0.49, *t* = 3.08, *p* = 0.004), with significant explanatory power (*R*^2^ = 0.24; *F*_(2,30)_ = 4.81; *p* = 0.015). The GI composite difference was a significant predictor of the FM functional status (*β* = 0.39, *t* = 2.27, *p* = 0.031) with an explanatory power trend (*R*^2^ = 0.15; *F*_(2,30)_ = 2.57; *p* = 0.094), and the CRP difference was a significant predictor of the FM functional status (*β* = 0.55, *t* = 3.39, *p* = 0.002) with a significant explanatory power (*R*^2^ = 0.28; *F*_(2,30)_ = 5.85; *p* = 0.007). No other effects of biomarkers on FM functional status were observed (Table 3). The stepwise regression analyses showed that both BDNF pre‒post change scores (*β* = 0.40; *t* = 2.75, *p* = 0.010) and CRP pre‒post change scores (*β* = 0.39; *t* = 2.69, *p* = 0.011) significantly explained the FM functional status pre‒follow up difference (*R*^2^ = 0.39; *F*_(2,30)_ = 9.60; *p* = 0.001), with no indications of collinearity (*T* = 0.95; *FIV* = 1.05).

**Table 3:** Direct and bootstrap indirect effects in the mediation/moderation models of biomarkers in FM functional status

Biomarkers	*R* ^2^	*F*_*(df)*_	*p* ^a^	Direct Effects					Indirect Effects			
				*path*	*Coeff*.	*SE*	*t*	*p* ^b^	*path*	*Boot*.	*SE*	*95% CI*
BDNF	0.24	4.81_(2, 30)_	0.015	*a*	2.81	1.24	2.26	0.031	*a x b*	5.24	3.48	0.24 to 13.68
				*b*	1.87	0.61	3.08	0.004				
				*c*′	1.90	4.56	0.42	0.680				
				*d*	−0.02	0.84	−0.02	0.983				
GI-comp	0.15	2.57 _(2, 30)_	0.094	*a*	0.27	0.18	1.48	0.148	*a x b*	2.70	2.36	−0.98 to 8.27
				*b*	10.07	4.45	2.27	0.031				
				*c*′	4.44	4.65	0.96	0.347				
				*d*	−1.48	3.85	−0.39	0.703				
CRP	0.28	5.85 _(2, 30)_	0.007	*a*	0.06	0.15	0.41	0.685	*a x b*	1.05	2.56	−5.53 to 4.97
				*b*	17.56	5.18	3.39	0.002				
				*c*′	6.09	4.13	1.47	0.151				
				*d*	−6.10	4.48	−1.36	0.183				
IL-6	0.02	0.27 _(2, 30)_	0.768	*a*	0.12	0.09	1.45	0.158	*a x b*	−0.26	1.80	−3.15 to 4.21
				*b*	−2.12	10.09	−0.21	0.835				
				*c*′	7.40	4.97	1.49	0.147				
				*d*	13.15	18.32	0.72	0.479				
TNF-α	0.01	0.09 _(2, 30)_	0.912	*a*	0.09	0.05	1.77	0.085	*a x b*	0.14	2.30	−3.39 to 6.22
				*b*	1.62	19.60	0.08	0.935				
				*c*′	7.00	5.13	1.37	0.182				
				*d*	5.96	14.30	0.42	0.680				
IL-10	0.03	0.49 _(2, 30)_	0.617	*a*	−0.04	0.02	−2.00	0.054	*a x b*	1.27	2.97	−4.70 to 6.05
				*b*	−36.19	60.62	−0.60	0.555				
				*c*′	5.87	5.23	1.12	0.271				
				*d*	17.16	65.43	0.26	0.795				

BDNF, GI-comp, CRP, IL-6, TNF-α and IL-10 difference scores were calculated as pre-post change scores (FIQ difference scores are pre-follow up change scores). Path coefficients are unstandardized ordinary least squares (OLS)-based regression coefficients. R^2^: determination coefficient (variance explained by regression models). F: Snedecor’s F associated with the adjustment of the regression model by using ANOVA. Coeff: unstandardized slope. t: Student’s t associated with the unstandardized slope by using the Wald test. SE: standard error. p^a^: p-value related to F-test. p^b^: p-value related to t-test. Boot: bootstrapped indirect effects using 10,000 samples. 95% CI: 95% confidence interval. “*a x b*” = indirect effects. *c*′ = direct effects adjusted by the mediating effect. *d* = moderating effect (see Fig. 2).

However, only BDNF showed significant indirect effects on FM functional status after calculating mediation/moderation path analyses (Table 3). It was observed that the repeated-measures factor (“X”) indirectly influenced the change in FM functional status at follow-up through its effects on BDNF pre‒post change scores. Participants at post-test exhibited improvements in BDNF (*a* = 2.81; *p* = 0.031), and this improvement in BDNF predicted an improvement in FM functional status at follow-up (*b* = 1.87; *p* = 0.004). A bias-corrected bootstrap confidence interval for the indirect effect (*a x b* = 5.24) based on 10,000 bootstrap samples was entirely above zero (95% CI = 0.24–12.25). There was no evidence that the repeated-measures factor influenced the change in FM functional status independent of its effects on BDNF (*c*′ = 1.90; *t* = 0.42, *p* = 0.680). In other words, the residual FM functional status pre‒follow up difference that was over and above the mediating effect (i.e. after centring the biomarker sum) was not significant, but the intercept was not completely null, and, therefore, partial mediation was suggested^61^.

## Discussion

Through the present pilot study, we have seen that the ABCT regimen of exercises managed to reduce FM functional impairment as well as levels of GI composite and CRP marker in FM patients compared to an active control condition based on relaxation practices with large effects. Interestingly, we found that reductions in GI composite and CRP inflammatory marker were significantly related and subsequently followed by a relief in the severity of FM symptoms, meaning that reductions in the levels of those biomarkers that are theoretically pro-inflammatory^8^ might work as mechanisms of change, facilitating improvements in FM functional status. However, both CRP and GI composite showed non-significant total indirect effects, maybe as a result of considering the total group as a whole to gain statistical power but at the cost of losing treatment specificity. Other RCTs evaluating the effects of mindfulness exercises on markers of inflammation found no significant reductions in healthy dementia caregivers^62^, but they found trends in university workers at risk for cardiovascular disease^28^ and in lonely elderly^27^. It has been suggested that inflammation biomarkers are more likely to be reduced by mind-body therapies such as meditation when they are implemented in populations with disease conditions rather than in healthy persons^63^. However, Rosenkranz *et al*.^29^ and Pace *et al*.^64^ found that mindfulness and compassion practices respectively reduced stress-induced immune and behavioural responses in healthy volunteers, and it was possibly through reduced stress reactivity learned through meditation exercises.

The biological pathways through which meditation could exert its benefits are not fully known, but they seem to include common and dissociable neural patterns with a relaxation response^65^. Considering that meditation may influence neural regions that might indirectly regulate stress^66^ ‒ including the hypothalamic-pituitary-adrenal axis (HPA)^67^ ‒ that the HPA might determine the production and inhibition of pro-inflammatory cytokines^62^ and that some of the pro-inflammatory cytokines are in turn potent activators of the HPA axis^68^, the idea that inflammatory responses could mediate changes related to the regulation of emotional and affective responses to stress, thereby inducing reductions in disease symptoms, seems to be plausible^69^. However, we cannot overlook the fact that inflammatory biomarkers are complex to analyse, as the variability among these molecules is linked to a very large number of components, ranging from behavioural to environmental and emotional to biological or medical factors. Moreover, we only evaluated three cytokines (IL-6, TNF-α, IL-10) and a non-specific inflammatory marker (CRP) as potential inflammatory biomarkers, even though there are plenty more molecules playing similar roles and interfering in the same processes already referenced^12,70^. In addition, we also let the patients keep their TAU, assuming that around half of them were taking antidepressants that are well-known to interact with the inflammatory system^71^. Even though we knew this was a risk that could alter results, we preferred to take it in order to provide this study with maximum external validity.

On the other hand, we have also observed that the ABCT treatment caused significant reductions in the BDNF levels of FM patients compared to the active control condition, which was based on relaxation exercises, with large effects. In addition, it was observed that reductions in BDNF were significantly related and followed by a decrease in FM functional impairment, showing significant indirect effects, which means that reductions in BDNF might function as an important ‒ but partial ‒ mechanism facilitating improvements in FM functional status. Several studies have suggested that FM and other central sensitivity syndromes present not only with elevated levels of general chronic inflammation but also with abnormalities in biomarkers related to neuronal plasticity, such as BDNF^7,72^. The BDNF baseline levels of the FM patients included in the present study were similar to those obtained in a previous study with patients suffering from central sensitivity syndrome with persistent somatic or visceral nociception^72.^ Interestingly, the BDNF levels of the ABCT group at post-test approached those of pain-free controls in the referenced study^72^, being that all of the BDNF values obtained in the present study fell within the range of possible values in human serum^73^. Although BDNF is generally known to have a neuroprotective role, circulating levels of BDNF have been found to be significantly elevated in FM compared with healthy controls^18^, although their associations with the patients’ clinical complaints remains elusive^16,71^ and some studies have found no differences between FM patients and healthy controls^74^. Nevertheless, similar results to those obtained in our work were reported in a study in which BDNF levels decreased after a 2-week thermal therapy programme, with significant pre‒post treatment decreases in BDNF accompanied by benefits in the general health status^75^.

In a previous study, ABCT demonstrated more efficacy than relaxation techniques for reducing FM clinical symptomatology^26^. The results of the present work extend those previous findings by analysing changes in the BDNF and inflammation biomarkers as a result of those interventions. However, we must bear in mind that this was a secondary analysis of a RCT, with the limitation that it had a small sample size on which several comparisons were explored, thereby increasing the possibility of type-I and type-II errors. In addition, although there were no statistically significant between-group differences in opioid consumption at baseline or post-test assessments, an opposite evolution in the number of consumers was observed throughout the RCT. The ABCT group reduced opioid consumption whereas the relaxation group increased consumption. This result, although non-significant, should not be overlooked, because opioids have well-known effects on the endocrine and immune systems. It has been observed using animal models that pain syndromes modulate activity in endogenous pain control circuits, and this effect is sympatho-adrenal-dependent^76^; opiate receptor antagonists, such as naloxone, alter pain-related effects of BDNF^77^; and chronic tramadol administration (an opioid for treating moderate to severe pain that was used the most by opioid consumers in our sample) can produce neurotoxic effects via inflammation increases, implicating cytokines such as IL-6 and TNF-α^78^. Epidemiological studies suggest there is a reciprocal interaction between the immune system and endogenous as well as exogenous opioids, but RCTs are needed to elucidate the specific role of the opioid-immune system interaction in patients to determine its clinical relevance^79^. It has also been observed that opiate consumption may produce neurotoxicity, modifying epigenetic processes that may be associated with peripheral alterations of BDNF, but the clinical implications of those changes are not clear, and more investigation is necessary^80^. Thus, in absence of clear hypotheses, future studies should investigate how opioid consumption might alter the modulation of endogenous pain control circuits and the production and pain-related effects of pro-inflammatory cytokines and BDNF in FM patients. Finally, we only conducted one blood collection at pre- and at post-treatment. Therefore, future research should use higher-powered designs, primarily aimed at assessing biomarker behaviour in FM patients to reduce possible errors related to multiple comparisons, by performing measurements on two sets of samples (e.g. serum and plasma) and ideally using two different methods (e.g. investigation of gene expression and protein levels), as is usually recommended^12,81^.

To the best of our knowledge, this is the first study to evaluate neurotrophic and inflammatory changes associated with the ABCT application in patients with FM. Thus, the results obtained in the present pilot RCT might serve as a heuristic force to conduct future studies of this third-wave psychological intervention, moving forward in a promising area of research. The enormous heterogeneity in the neurotrophic response and inflammatory systems adds special challenges to this area. That said, in summation, this preliminary work leads us to suggest that, as hypothesized, neurotrophic factor and inflammation levels seem to be reduced by the ABCT exercises in FM patients and that they might be mechanisms of FM functional status improvement. These findings are in line with both the idea that alterations in central nervous system nociceptive processing are present in people with FM and that a potential mechanism might include a peripheral nervous system component associated with the generation of pain, with a possible role of systemic inflammation^6,69^. Contrary to previous proposals that intended to explain the heterogeneity of FM clinical responses by conferring a moderating role to inflammatory biomarkers^20,54^, we did not find that role for either BDNF or the inflammatory markers used in the present work. In this sense, it has also been pointed out that some of the heterogeneous responses to FM treatment could be due to differences in the predominance of the referenced mechanisms underlying a peripherally driven pain condition involving low-grade systemic inflammation or to alterations of the central nervous system involved in nociceptive pain processing^6,69^.

A general hypothesis that could frame our findings may be that ABCT takes root in a motivational system that encourages an individual to re-contextualize their life by accepting their circumstances and by improving their attachment style and positive and compassionate engagement with specific aspects of their life. This may be achieved through awareness and clarification of personal values in family, social relationships, work, and personal development^26,31^. All of this might, in turn, reduce the impact of psychological factors that provoke stress and emotional reactivity and enhance the pain experience, thereby benefiting the regulation of BDNF and inflammatory status, stimulating commitment to valued actions, and favouring an improvement in FM health status and symptomatology^82^. Nevertheless, future research with higher-powered designs should investigate the specific sequence of these events as well as whether changes in mindfulness, self-compassion and psychological flexibility, on the one hand, and neurotrophic and inflammatory responses as well as FM symptoms, on the other hand, are conditioned and linked to each other as a result of the ABCT practices.

## Supplementary information

Dataset 1

## Data Availability

The datasets generated during and/or analysed during the current study are available in the OSF repository, https://osf.io/vqyd6/.
